# Decidable Synthesis of Programs with Uninterpreted Functions

**DOI:** 10.1007/978-3-030-53291-8_32

**Published:** 2020-06-16

**Authors:** Paul Krogmeier, Umang Mathur, Adithya Murali, P. Madhusudan, Mahesh Viswanathan

**Affiliations:** 8 Microsoft Research Lab, Redmond, WA USA; 9 University of Southern California, Los Angeles, CA USA; University of Illinois at Urbana-Champaign, Champaign, USA

## Abstract

We identify a decidable synthesis problem for a class of programs of unbounded size with conditionals and iteration that work over infinite data domains. The programs in our class use uninterpreted functions and relations, and abide by a restriction called coherence that was recently identified to yield decidable verification. We formulate a powerful grammar-restricted (syntax-guided) synthesis problem for coherent uninterpreted programs, and we show the problem to be decidable, identify its precise complexity, and also study several variants of the problem.

## Introduction

Program synthesis is a thriving area of research that addresses the problem of automatically constructing a program that meets a user-given specification [1, 21, 22]. Synthesis specifications can be expressed in various ways: as input-output examples [19, 20], temporal logic specifications for reactive programs [44], logical specifications [1, 4], etc. Many targets for program synthesis exist, ranging from transition systems [31, 44], logical expressions [1], imperative programs [51], distributed transition systems/programs [38, 43, 45], filling holes in programs [51], or repairs of programs [49].

A classical stream of program synthesis research is one that emerged from a problem proposed by Church [13] in 1960 for Boolean circuits. Seminal results by Büchi and Landweber [9] and Rabin [48] led to a mature understanding of the problem, including connections to infinite games played on finite graphs and automata over infinite trees (see [18, 32]). Tractable synthesis for temporal logics like LTL, CTL, and their fragments was investigated and several applications for synthesizing hardware circuits emerged [6, 7].

In recent years, the field has taken a different turn, tackling synthesis of programs that work over infinite domains such as strings [19, 20], integers [1, 51], and heaps [47]. Typical solutions derived in this line of research involve (a) bounding the class of programs to a finite set (perhaps iteratively increasing the class) and (b) searching the space of programs using techniques like symmetry-reduced enumeration, SAT solvers, or even random walks [1, 4], typically guided by counterexamples (CEGIS) [28, 34, 51]. Note that iteratively searching larger classes of programs allows synthesis engines to find a program if one exists, but it does not allow one to conclude that there is no program that satisfies the specification. Consequently, in this stream of research, decidability results are uncommon (see Sect. 7 for some exceptions in certain heavily restricted cases).

*In this paper we present, to the best of our knowledge, the first decidability results for program synthesis over a natural class of programs with iteration/recursion, having arbitrary sizes, and which work on infinite data domains. In particular, we show decidable synthesis of a subclass of programs that use uninterpreted functions and relations.*

Our primary contribution is a decidability result for realizability and synthesis of a restricted class of imperative *uninterpreted* programs. Uninterpreted programs work over infinite data models that give arbitrary meanings to their functions and relations. Such programs satisfy their assertions if they hold along all executions for *every* model that interprets the functions and relations. The theory of uninterpreted functions and relations is well studied—classically, in 1929, by Gödel, where completeness results were shown [5] and, more recently, its decidable quantifier-free fragment has been exploited in SMT solvers in combination with other theories [8]. In recent work [39], a subclass of uninterpreted programs, called *coherent* programs, was identified and shown to have a decidable verification problem. Note that in this verification problem there are no user-given loop invariants; the verification algorithm finds inductive invariants and proves them automatically in order to prove program correctness.

In this paper, we consider the synthesis problem for coherent uninterpreted programs. The user gives a *grammar* $\mathcal {G}$ that generates well-formed programs in our programming language. The grammar can force programs to have $\mathbf{assert}$ statements at various points which collectively act as the specification. The program synthesis problem is then to construct a coherent program, if one exists, conforming to the grammar $\mathcal {G}$ that satisfies all assertions in all executions when running on *any* data model that gives meaning to function and relation symbols.

Our primary result is that the realizability problem (checking the existence of a program conforming to the grammar and satisfying its assertions) is decidable for coherent uninterpreted programs. We prove that the problem is $2\mathsf {EXPTIME}$-complete. Further, whenever a correct coherent program that conforms to the grammar exists, we can synthesize one. We also show that the realizability/synthesis problem is undecidable if the coherence restriction is dropped. In fact we show a stronger result that the problem is undecidable even for synthesis of *straight-line* programs (without conditionals and iteration)!

Coherence of programs is a technical restriction that was introduced in [39]. It consists of two properties, both of which were individually proven to be essential for ensuring that program verification is decidable. Intuitively, the restriction demands that functions are computed on any tuple of terms only once and that assumptions of equality come early in the executions. In more recent work [41], the authors extend this decidability result to handle map updates, and applied it to memory safety verification for a class of heap-manipulating programs on forest data-structures, demonstrating that the restriction of coherence is met in practice by certain natural and useful classes of programs.

Note that automatic synthesis of correct programs over infinite domains demands that we, at the very least, can automatically verify the synthesized program to be correct. The class of coherent uninterpreted programs identified in the work of [39] is the only natural class of programs we are aware of that has recursion and conditionals, works over infinite domains, and admits decidable verification. Consequently, this class is a natural target for proving a decidable synthesis result.

The problem of synthesizing a program from a grammar with assertions is a powerful formulation of program synthesis. In particular, the grammar can be used to restrict the space of programs in various ways. For example, we can restrict the space syntactically by disallowing while loops. Or, for a fixed *n*, by using a set of Boolean variables linear in *n* and requiring a loop body to strictly increment a counter encoded using these variables, we can demand that loops terminate in a linear/polynomial/exponential number of iterations. We can also implement loops that do not always terminate, but terminate only when the data model satisfies a particular property, e.g., programs that terminate only on finite list segments, by using a skeleton of the form:  . Grammar-restricted program synthesis can express the synthesis of programs with holes, used in systems like Sketch [50], where the problem is to fill holes using programs/expressions conforming to a particular grammar so that the assertions in the program hold. Synthesizing programs or expressions using restricted grammars is also the cornerstone of the intensively studied SyGuS (syntax-guided synthesis) format [1, 52]^1^.

The proof of our decidability result relies on tree automata, a callback to classical theoretical approaches to synthesis. The key idea is to represent programs as trees and build automata that accept trees corresponding to correct programs. The central construction is to build a two-way alternating tree automaton that accepts *all* program trees of coherent programs that satisfy their assertions. Given a grammar $\mathcal {G}$ of programs (which has to satisfy certain natural conditions), we show that there is a regular set of program trees for the language of allowed programs $L(\mathcal {G})$. Intersecting the automata for these two regular tree languages and checking for emptiness establishes the upper bound. Our constructions crucially use the automaton for verifying coherent uninterpreted programs in [39] and adapt ideas from [35] for building two-way automata over program trees. Our final decision procedure is doubly-exponential in the number of program variables and  in the size of the grammar. We also prove a matching lower bound by reduction from the acceptance problem for alternating exponential-space Turing machines. The reduction is non-trivial in that programs (which correspond to runs in the Turing machine) must simulate sequences of configurations, each of which is of exponential size, by using only polynomially-many variables.

**Recursive Programs, Transition Systems, and Boolean Programs:** We study three related synthesis problems. First, we show that our results extend to synthesis of call-by-value *recursive* uninterpreted programs (with a fixed number of functions and fixed number of local/global variables). This problem is also $2\mathsf {EXPTIME}$-complete but is more complex, as even single executions simulated on the program tree must be split into separate copies, with one copy executing the summary of a function call and the other proceeding under the assumption that the call has returned in a summarized state.

We next examine a synthesis problem for *transition systems*. Transition systems are similar to programs in that they execute similar kinds of atomic statements. We allow the user to restrict the set of allowable executions (using regular sets). Despite the fact that this problem seems very similar to program synthesis, we show that it is an *easier* problem, and coherent transition system realizability and synthesis can be solved in time exponential in the number of program variables and polynomial in the size of the automata that restrict executions. We prove a corresponding lower bound to establish $\mathsf {EXPTIME}$-completeness of this problem.

Finally, we note that our results also show, as a corollary, that the grammar-restricted realizability/synthesis problem for Boolean programs (resp. execution-restricted synthesis problem for Boolean transition systems) is decidable and is $2\mathsf {EXPTIME}$-complete (resp. $\mathsf {EXPTIME}$-complete). These results for Boolean programs are themselves new. The lower bound results for these problems hence show that coherent program/transition-system synthesis is not particularly harder than Boolean program synthesis for uninterpreted programs. Grammar-restricted Boolean program synthesis is an important problem which is addressed by many practical synthesis systems like Sketch [50].

Due to space restrictions, we present only proof gists for main results in the paper. All the complete proofs can be found in our technical report [30].

## Examples

We will begin by looking at several examples to gain some intuition for uninterpreted programs.

### Example 1

**Fig. 1.**
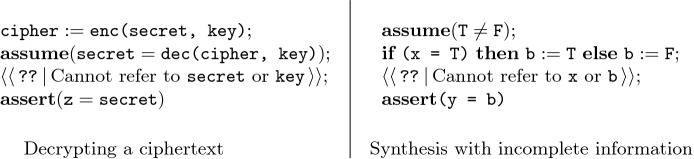
Examples of programs with holes

Consider the program in Fig. 1 (left). This program has a *hole* ‘’ that we intend to fill with a sub-program so that the entire program (together with the contents of the hole) satisfies the assertion at the end. The sub-program corresponding to the hole is allowed to use the variable cipher as well as some additional variables $\texttt {y}_1, \ldots , \texttt {y}_n$ (for some fixed *n*), but is not allowed to refer to $\texttt {key}$ or $\texttt {secret}$ in any way. Here we also restrict the hole to exclude while loops. This example models the encryption of a secret message $\texttt {secret}$ with a key $\texttt {key}$. The assumption in the second line of the program models the fact that the secret message can be decrypted from $\texttt {cipher}$ and $\texttt {key}$. Here, the functions $\texttt {enc}$ and $\texttt {dec}$ are *uninterpreted functions*, and thus the program we are looking for is an *uninterpreted program*. For such a program, the assertion $\text {``}\mathbf{assert} (\texttt {z} = \texttt {secret})\text {''}$ holds at the end if it holds for *all models*, i.e, for all interpretations of enc and dec and for all initial values of the program variables $\texttt {secret}$, $\texttt {key}$, $\texttt {cipher}$, and $\texttt {y}_1, \ldots , \texttt {y}_n$. With this setup, we are essentially asking whether a program that does not have access to $\texttt {key}$ can recover $\texttt {secret}$. It is not hard to see that there is no program which satisfies the above requirement. The above modeling of keys, encryption, nonces, etc. is common in algebraic approaches to modeling cryptographic protocols [15, 16].

### Example 2

The program in Fig. 1 (right) is another simple example of an unrealizable specification. The program variables here are $\texttt {x}, \texttt {b}$, and $\texttt {y}$. The hole in this partial program is restricted so that it cannot refer to $\texttt {x}$ or $\texttt {b}$. It is easy to phrase the question for synthesis of the complete program in terms of a grammar. The restriction on the hole ensures that the synthesized code fragment can neither directly check if $\texttt {x} = \texttt {T}$, nor indirectly check via $\texttt {b}$. Consequently, it is easy to see that there is no program for the hole that can ensure $\texttt {y}$ is equal to $\texttt {b}$. We remark that the code at the hole, apart from not being allowed to examine some variables, is also implicitly prohibited from looking at the control path taken to reach the hole. If we could synthesize two different programs depending on the control path taken to reach the hole, then we could set $\texttt {y} :=\texttt {T}$ when the **then**-branch is taken and set $\texttt {y} :=\texttt {F}$ when the **else**-branch is taken. Program synthesis requires a control-flow independent decision to be made about how to fill the hole. In this sense, we can think of the hole as having only *incomplete information* about the executions for which it must be correct. This can be used to encode specifications using complex ghost code, as we show in the next examples. In Sect. 6, we explore a slightly different synthesis problem, called *transition system synthesis*, where holes can be differently instantiated based on the history of an execution.

### Example 3

In this example, we model the synthesis of a program that checks whether a linked list pointed to by some node x has a key k. We model a $\textit{next}$ pointer with a unary function next and we model locations using elements in the underlying data domain.

**Fig. 2.**
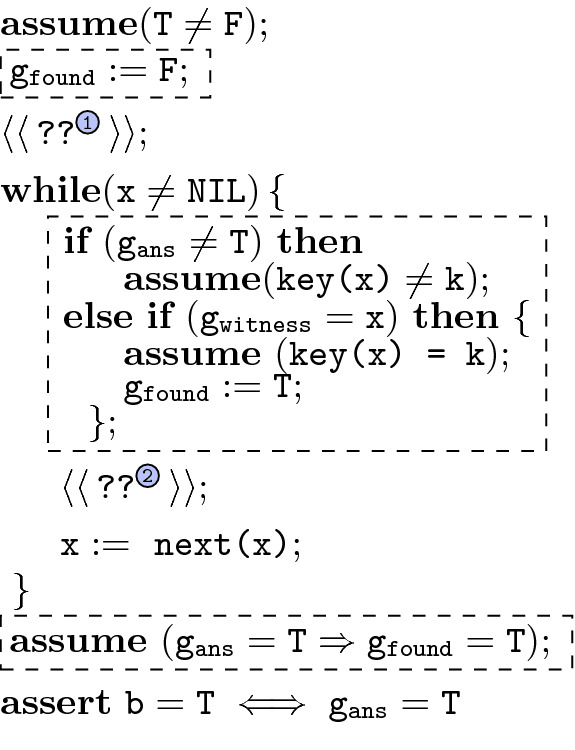
Skeleton with ghost code

Our formalism allows only for $\mathbf{assert}$ statements to specify desired program properties. In order to state the correctness specification for our desired list-search program, we interleave *ghost code* into the program skeleton; we distinguish ghost code fragments by enclosing them in  . The skeleton in Fig. 2 has a loop that advances the pointer variable x along the list until NIL is reached. We model NIL with an immutable program variable. The first hole ‘’ before the **while**-loop and the second hole ‘’ within the **while**-loop need to be filled so that the assertion at the end is satisfied. We use three ghost variables in the skeleton: $\mathtt {g_{ans}}$, $\mathtt {g_{witness}}$, and $\mathtt {g_{found}}$. The ghost variable $\mathtt {g_{ans}}$ evaluates to whether we expect to find $\texttt {k}$ in the list or not, and hence at the end the skeleton asserts that the Boolean variable $\texttt {b}$ computed by the holes is precisely $\mathtt {g_{ans}}$. The holes are restricted to not look at the ghost variables.

Now, notice that the skeleton needs to *check* that the answer $\mathtt {g_{ans}}$ is indeed correct. If $\mathtt {g_{ans}}$ is not $\texttt {T}$, then we add the assumption that $\texttt {key(x)} \ne \texttt {k}$ in each iteration of the loop, hence ensuring the key is not present. For ensuring correctness in the case $\mathtt {g_{ans}}= \texttt {T}$, we need two more ghost variables $\mathtt {g_{witness}}$ and $\mathtt {g_{found}}$. The variable $\mathtt {g_{witness}}$ witnesses the precise location in the list that holds the key $\texttt {k}$, and variable $\mathtt {g_{found}}$ indicates whether the location at $\mathtt {g_{witness}}$ belongs to the list pointed to by $\texttt {x}$. Observe that this specification can be realized by filling ‘’ with “$\texttt {b} :=\texttt {F}$” and ‘’ with “$\mathbf{if} ~ \texttt {key(x)} = \texttt {k} ~\mathbf{then} ~ \texttt {b} :=\texttt {T}$”, for instance. Furthermore, this program is *coherent* [39] and hence our decision procedure will answer in the affirmative and synthesize code for the holes.

In fact, our procedure will synthesize a representation for *all* possible ways to fill the holes (thus including the solution above) and it is therefore possible to enumerate and pick specific solutions. It is straightforward to formulate a grammar which matches this setup. As noted, we must stipulate that the holes do not use the ghost variables.

### Example 4

Consider the same program skeleton as in Example 3, but let us add an assertion at the end: “$\mathbf{assert} ~(\texttt {b = T} \Rightarrow \texttt {z}=\mathtt {g_{witness}})$”, where $\texttt {z}$ is another program variable. We are now demanding that the synthesized code also find a location $\texttt {z}$, whose key is $\texttt {k}$, that is equal to the ghost location $\mathtt {g_{witness}}$, which is guessed nondeterministically at the beginning of the program. This specification is *unrealizable*: for a list with multiple locations having the key $\texttt {k}$, no matter what the program picks we can always take $\mathtt {g_{witness}}$ to be the *other* location with key $\texttt {k}$ in the list, thus violating the assertion. Our decision procedure will report in the negative for this specification.

### Example 5 (Input/Output Examples)

**Fig. 3.**
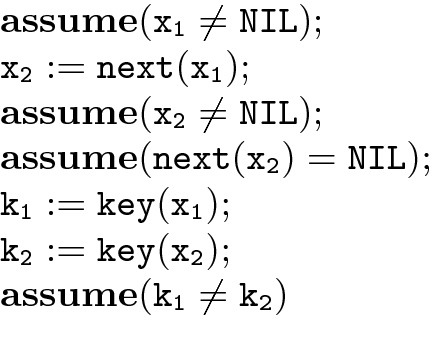
An example model

We can encode input/output examples by adding a sequence of assignments and assumptions that define certain models at the beginning of the program grammar. For instance, the sequence of statements in Fig. 3 defines a linked list of two elements with different keys.

We can similarly use special variables to define the output that we expect in the case of each model. And as we saw in the ghost code of Fig. 2, we can use fresh variables to introduce nondeterministic choices, which the grammar can use to pick an example model nondeterministically. Thus when the synthesized program is executed on the chosen model it computes the expected answer. This has the effect of requiring a solution that generalizes across models. See [30] for a more detailed example.

## Preliminaries

In this section we define the syntax and semantics of uninterpreted programs and the *(grammar-restricted) uninterpreted program synthesis* problem.

**Syntax.** We fix a first order signature $\varSigma = (\mathcal {F}, \mathcal {R})$, where $\mathcal {F}$ and $\mathcal {R}$ are sets of function and relation symbols, respectively. Let *V* be a finite set of program variables. The set of programs over *V* is inductively defined using the following grammar, with $f \in \mathcal {F}$, $R \in \mathcal {R}$ (with *f* and *R* of the appropriate arities), and $x, y, z_1, \ldots , z_r \in V$.

$$\begin{aligned} \langle stmt \rangle _V~{:}{:}\!\!=\,\,&\,\, \mathbf{skip} \, \mid \, x :=y \, \mid \, x :=f(z_1, \ldots , z_r) \, \mid \, \\&\mathbf{assume} \, \big (\langle cond \rangle _V\big ) \, \mid \, \mathbf{assert} \, \big (\langle cond \rangle _V\big ) \mid \, \langle stmt \rangle _V \, ;\, \langle stmt \rangle _V \mid \, \\&\mathbf{if} \, \big (\langle cond \rangle _V\big ) \, \mathbf{then} \, \langle stmt \rangle _V \, \mathbf{else} \, \langle stmt \rangle _V \, \mid \, \mathbf{while} \, \big (\langle cond \rangle _V\big ) \, \langle stmt \rangle _V \\ \langle cond \rangle _V~{:}{:}\!\!=\,\,&\, x = y \, \mid \, R(z_1, \ldots , z_r) \, \mid \, \langle cond \rangle _V \vee \langle cond \rangle _V \, \mid \, \lnot \langle cond \rangle _V \end{aligned}$$

Without loss of generality, we can assume that our programs do not use relations (they can be modeled with functions) and that every condition is either an equality or disequality between variables (arbitrary Boolean combinations can be modeled with nested $\mathbf{if} {-}\mathbf{then} {-}\mathbf{else}$). When the set of variables *V* is clear from context, we will omit the subscript *V* from $\langle stmt \rangle _V$ and $\langle cond \rangle _V$.

**Program Executions.** An execution over *V* is a finite word over the alphabet

$$\begin{aligned} \varPi _V = \{ \text {``}x :=y\text {''}, \text {``}x :=f(\overline{z})\text {''},&\text {``}\mathbf{assume} (x = y)\text {''}, \text {``}\mathbf{assume} (x \ne y)\text {''}, \\&\text {``}\mathbf{assert} (\bot )\text {''} \,\mid \, x, y\in V, \overline{z}\in V^r, f \in \mathcal {F}\}. \end{aligned}$$

The set of *complete executions* for a program *p* over *V*, denoted $\textsf {Exec}(p)$, is a regular language. See [30] for a straightforward definition. The set $\textsf {PExec}(p)$ of *partial executions* is the set of prefixes of complete executions in $\textsf {Exec}(p)$. We refer to partial executions as simply *executions*, and clarify as needed when the distinction is important.

**Semantics.** The semantics of executions is given in terms of data models. A data model $\mathcal {M}= (U, \mathcal {I})$ is a first order structure over $\varSigma$ comprised of a universe *U* and an interpretation function $\mathcal {I}$ for the program symbols. The semantics of an execution $\pi$ over a data model $\mathcal {M}$ is given by a configuration $\sigma (\pi , \mathcal {M}) : V \rightarrow U$ which maps each variable to its value in the universe *U* at the end of $\pi$. This notion is straightforward and we skip the formal definition (see [39] for details). For a fixed program *p*, any particular data model corresponds to at most one complete execution $\pi \in \textsf {Exec}(p)$.

An execution $\pi$ is *feasible* in a data model $\mathcal {M}$ if for every prefix $\rho = \rho ' \cdot \mathbf{assume} (x \sim y)$ of $\pi$ (where $\sim \,\in \{=,\ne \}$), we have $\sigma (\rho ', \mathcal {M})(x) \sim \sigma (\rho ', \mathcal {M})(y)$. Execution $\pi$ is said to be *correct* in a data model $\mathcal {M}$ if for every prefix of $\pi$ of the form $\rho = \rho ' \cdot \mathbf{assert} (\bot )$, we have that $\rho '$ is not feasible, or *infeasible* in $\mathcal {M}$. Finally, a program *p* is said to be *correct* if for all data models $\mathcal {M}$ and executions $\pi \in \textsf {PExec}(p)$, $\pi$ is correct in $\mathcal {M}$.

### The Program Synthesis Problem

We are now ready to define the program synthesis problem. Our approach will be to allow users to specify a grammar and ask for the synthesis of a program from the grammar. We allow the user to express specifications using *assertions* in the program to be synthesized.

**Grammar Schema and Input Grammar.** In our problem formulation, we allow users to define a grammar which conforms to a schema, given below. The input grammars allow the usual context-free power required to describe proper nesting/bracketing of program expressions, but disallow other uses of the context-free power, such as *counting statements*.

**Fig. 4.**
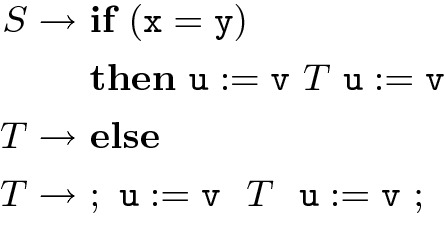
Grammar with counting

For example, we disallow the grammar in Fig. 4. This grammar has two non-terminals *S* (the start symbol) and *T*. It generates programs with a conditional that has the *same* number of assignments in the $\mathbf{if}$ and $\mathbf{else}$ branches. We assume a countably infinite set $PN$ of nonterminals and a countably infinite set $PV$ of program variables. The grammar schema $\mathcal {S}$ over $PN$ and $PV$ is an infinite collection of productions:An *input grammar* $\mathcal {G}$ is any finite subset of the schema $\mathcal {S}$, and it defines a set of programs, denoted $L(\mathcal {G})$. We can now define the main problem addressed in this work.

#### Definition 1 (Uninterpreted Program Realizability and Synthesis)

Given an input grammar $\mathcal {G}$, the realizability problem is to determine whether there is an uninterpreted program $p \in L(\mathcal {G})$ such that *p* is correct. The synthesis problem is to determine the above, and further, if realizable, synthesize a correct program $p \in L(\mathcal {G})$.

#### Example 6

Consider the program with a hole from Example 1 (Fig. 1, left). We can model that synthesis problem in our framework with the following grammar.

$$\begin{aligned}&\begin{array}{ll} S \rightarrow P_1; P_2; P_{\langle \langle \,\texttt {??}^{}\, \rangle \rangle }; P_3 &{} P_{\langle \langle \,\texttt {??}^{}\, \rangle \rangle } \rightarrow \langle stmt \rangle _{V_{\langle \langle \,\texttt {??}^{}\, \rangle \rangle }} \\ P_1 \rightarrow \text {``}\texttt {cipher} :=\texttt {enc(secret, key)}\text {''} &{} P_3 \rightarrow \text {``}\mathbf{assert} (\texttt {z} = \texttt {secret})\text {''} \\ P_2 \rightarrow \text {``}\mathbf{assume} (\texttt {secret} = \texttt {dec(cipher, key)})\text {''} &{} \end{array} \end{aligned}$$

Here, $V_{\langle \langle \,\texttt {??}^{}\, \rangle \rangle } = \{\texttt {cipher}, \texttt {y}_1, \ldots , \texttt {y}_n\}$ and the grammar $\langle stmt \rangle _{V_{\langle \langle \,\texttt {??}^{}\, \rangle \rangle }}$ is that of Sect. 3, restricted to loop-free programs. Any program generated from this grammar indeed matches the template from Fig. 1 (left) and any such program is correct if it satisfies the last assertion for all models, i.e., all interpretations of the function symbols enc and dec and for all initial values of the variables in $V = V_{\langle \langle \,\texttt {??}^{}\, \rangle \rangle } \cup \{\texttt {key}, \texttt {secret}\}$.

## Undecidability of Uninterpreted Program Synthesis

Since verification of uninterpreted programs with loops is undecidable [39, 42], the following is immediate.

### Theorem 1

The uninterpreted program synthesis problem is undecidable.

We next consider synthesizing loop-free uninterpreted programs (for which verification reduces to satisfiability of quantifier-free EUF) from grammars conforming to the following schema:

$$\mathcal {S}_\text {loop-free} = \mathcal {S}\setminus \{\text {``}P \rightarrow \, \mathbf{while} \, (x \sim y) \,\, P_1\text {''} \,\mid \, P, P_1 \in PN, \ x, y\in PV, \sim \,\in \{=, \ne \} \} $$

### Theorem 2

The uninterpreted program synthesis problem is undecidable for the schema $\mathcal {S}_\text {loop-free}$.

This is a corollary of the following stronger result: synthesis of *straight-line uninterpreted programs* (conforming to schema $\mathcal {S}_\textsf {SLP}$ below) is undecidable.

$$\begin{aligned} \mathcal {S}_\textsf {SLP}= \mathcal {S}_\text {loop-free} \setminus \{ \text {``}P \rightarrow \, \mathbf{if} (x\sim y) \, \mathbf{then} \, P_1 \, \mathbf{else} \, P_2\text {''} \,\,|\,\,&P, P_1, P_2 \in PN, \\&x, y \in PV, \,\sim \,\in \{=, \ne \} \} \end{aligned}$$

### Theorem 3

The uninterpreted program synthesis problem is undecidable for the schema $\mathcal {S}_\textsf {SLP}$.

In summary, program synthesis of even straight-line uninterpreted programs, which have neither conditionals nor iteration, is already undecidable. The notion of *coherence* for uninterpreted programs was shown to yield decidable verification in [39]. As we’ll see in Sect. 5, restricting to coherent programs yields decidable synthesis, even for programs with conditionals *and* iteration.

## Synthesis of Coherent Uninterpreted Programs

In this section, we present the main result of the paper: grammar-restricted program synthesis for uninterpreted *coherent* programs [39] is decidable. Intuitively, coherence allows us to maintain congruence closure in a streaming fashion when reading a coherent execution. First we recall the definition of coherent executions and programs in Sect. 5.1 and also the algorithm for verification of such programs. Then we introduce the synthesis procedure, which works by constructing a two-way alternating tree automaton. We briefly discuss this class of tree automata in Sect. 5.2 and recall some standard results. In Sects. 5.3, and 5.5 we describe the details of the synthesis procedure, argue its correctness, and discuss its complexity. In Sect. 5.6, we present a tight lower bound result.

### Coherent Executions and Programs

The notion of coherence for an execution $\pi$ is defined with respect to the *terms* it computes. Intuitively, at the beginning of an execution, each variable $x \in V$ stores some constant term $\widehat{x} \in \mathcal {C}$. As the execution proceeds, new terms are computed and stored in variables. Let $\textsf {Terms}_\varSigma$ be the set of all ground terms defined using the constants and functions in $\varSigma$. Formally, the term corresponding to a variable $x \in V$ at the end of an execution $\pi \in \varPi _V^*$, denoted $\textsf {T}(\pi , x) \in \textsf {Terms}_\varSigma$, is inductively defined as follows. We assume that the set of constants $\mathcal {C}$ includes a designated set of *initial* constants $\widehat{V} = \{\widehat{x} \,\mid \, x \in V\} \subseteq \mathcal {C}$.We will use $\textsf {T}(\pi )$ to denote the set $\{\textsf {T}(\pi ', x) \,\mid \, x \in V, \pi ' \text { is a prefix of } \pi \}$.

A related notion is the set of *term equality assumptions* that an execution accumulates, which we formalize as $\alpha : \pi \rightarrow \mathcal {P}(\textsf {Terms}_\varSigma \times \textsf {Terms}_\varSigma )$, and define inductively as $\alpha (\varepsilon ) = \varnothing$, $\alpha (\pi {\cdot }\text {``}\mathbf{assume} (x = y)\text {''}) = \alpha (\pi ) \cup \{(\textsf {T}(\pi , x), \textsf {T}(\pi , y))\}$, and $\alpha (\pi {\cdot }a) = \alpha (\pi )$ otherwise.

For a set of term equalities $A \subseteq \textsf {Terms}_\varSigma \times \textsf {Terms}_\varSigma$, and two ground terms $t_1, t_2 \in \textsf {Terms}_\varSigma$, we say $t_1$ and $t_2$ are *equivalent modulo* *A*, denoted $t_1 \cong _A t_2$, if $A \models t_1 = t_2$. For a set of terms $S \subseteq \textsf {Terms}_\varSigma$, and a term $t \in \textsf {Terms}_\varSigma$ we write $t \in _{A} S$ if there is a term $t' \in S$ such that $t \cong _{A} t'$. For terms $t, s \in \textsf {Terms}_\varSigma$, we say *s* is a *superterm modulo* *A* of *t*, denoted $t \preccurlyeq _{A} s$ if there are terms $t', s' \in \textsf {Terms}_\varSigma$ such that $t \cong _A t'$, $s \cong _A s'$ and $s'$ is a superterm of $t'$.

With the above notation in mind, we now review the notion of coherence.

#### Definition 2

**(Coherent Executions and Programs** [39]**).** An execution $\pi \in \varPi _V^*$ is said to be *coherent* if it satisfies the following two conditions.

- **Memoizing.** Let $\rho = \rho ' \cdot \text {``}x :=f(\overline{y})\text {''}$ be a prefix of $\pi$. If $t_x = \textsf {T}(\rho , x) \in _{\alpha (\rho ')} \textsf {T}(\rho ')$, then there is a variable $z \in V$ such that $t_x \cong _{\alpha (\rho ')} t_z$, where $t_z = \textsf {T}(\rho ', z)$.
- **Early Assumes.** Let $\rho = \rho ' \cdot \text {``}\mathbf{assume} (x = y)\text {''}$ be a prefix of $\pi$, $t_x = \textsf {T}(\rho ', x)$ and $t_y = \textsf {T}(\rho ', y)$. If there is a term $s \in \textsf {T}(\rho ')$ such that either $t_x \preccurlyeq _{\alpha (\rho ')} s$ or $t_y \preccurlyeq _{\alpha (\rho ')} s$, then there is a variable $z \in V$ such that $s \cong _{\alpha (\rho ')} t_z$, where $t_z = \textsf {T}(\rho ', z)$.

A program *p* is coherent if every complete execution $\pi \in \textsf {Exec}(p)$ is coherent.

The following theorems due to [39] establish the decidability of verifying coherent programs and also of checking if a program is coherent.

#### Theorem 4

**(**[39]**).** The verification problem for coherent programs, i.e. checking if a given uninterpreted coherent program is correct, is decidable.

#### Theorem 5

**(**[39]**).** The problem of checking coherence, i.e. checking if a given uninterpreted program is coherent, is decidable.

The techniques used in [39] are automata theoretic. They allow us to construct an automaton 2, of size $O(2^{\text {poly}(|V|)})$, which accepts all coherent executions that are also correct.

To give some intuition for the notion of coherence, we illustrate simple example programs that are not coherent. Consider program $p_0$ below, which is not coherent because it fails to be memoizing.

$$\begin{aligned} p_0\quad \overset{\Delta }{=}\quad \texttt {x} :=\texttt {f(y)};\, \texttt {x} :=\texttt {f(x)};\, \texttt {z} :=\texttt {f(y)} \end{aligned}$$

The first and third statements compute $f(\widehat{y})$, storing it in variables *x* and *z*, respectively, but the term is *dropped* after the second statement and hence is not contained in any program variable when the third statement executes. Next consider program $p_1$, which is not coherent because it fails to have early assumes.

$$\begin{aligned} p_1\quad \overset{\Delta }{=}\quad \texttt {x} :=\texttt {f(w)};\, \texttt {x} :=\texttt {f(x)}; \texttt {y} :=\texttt {f(z)};\, \texttt {y} :=\texttt {f(y)};\, \mathbf{assume} \texttt {(w = z)} \end{aligned}$$

Indeed, the assume statement is not early because superterms of *w* and *z*, namely $f(\widehat{w})$ and $f(\widehat{z})$, were computed and subsequently dropped before the assume.

Intuitively, the coherence conditions are necessary to allow equality information to be tracked with finite memory. We can make this stark by tweaking the example for $p_1$ above as follows.

$$\begin{aligned} p'_1\quad \overset{\Delta }{=}\quad&\texttt {x} :=\texttt {f(w)};\,\underbrace{\texttt {x}:=\texttt {f(x)}\cdots \texttt {x}:=\texttt {f(x)}}_{n\,\, \text {times}}; \\&\texttt {y} :=\texttt {f(z)};\,\underbrace{\texttt {y}:=\texttt {f(y)}\cdots \texttt {y}:=\texttt {f(y)}}_{n\,\, \text {times}};\, \mathbf{assume} \texttt {(w = z)} \end{aligned}$$

Observe that, for large *n* (e.g. $n>100$), many terms are computed and dropped by this program, like $f^{42}(\widehat{x})$ and $f^{99}(\widehat{y})$ for instance. The difficulty with this program, from a verification perspective, is that the assume statement entails equalities between many terms which have not been kept track of. Imagine trying to verify the following program

$$\begin{aligned} p_2 \quad \overset{\Delta }{=}\quad p'_1;\, \mathbf{assert} \texttt {(x = y)} \end{aligned}$$

Let $\pi _{p'_1}\in \textsf {Exec}(p'_1)$ be the unique complete execution of $p'_1$. If we examine the details, we see that $t_x = \textsf {T}(\pi _{p'_1},x) = f^{101}(\widehat{w})$ and $t_y = \textsf {T}(\pi _{p'_1},y) = f^{101}(\widehat{z})$. The assertion indeed holds because $t_x \cong _{\{(\widehat{w},\widehat{z})\}} t_y$. However, to keep track of this fact requires remembering an arbitrary number of terms that grows with the size of the program. Finally, we note that the coherence restriction is met by many single-pass algorithms, e.g. searching and manipulation of lists and trees.

### Overview of the Synthesis Procedure

Our synthesis procedure uses tree automata. We consider tree representations of programs, or *program trees*. The synthesis problem is thus to check if there is a program tree whose corresponding program is coherent, correct, and belongs to the input grammar $\mathcal {G}$.

The synthesis procedure works as follows. We first construct a top-down tree automaton  that accepts the set of trees corresponding to the programs generated by $\mathcal {G}$. We next construct another tree automaton , which accepts all trees corresponding to programs that are  oherent and  orrect.  is a two-way alternating tree automaton that simulates all executions of an input program tree and checks that each is both correct and coherent. In order to simulate longer and longer executions arising from constructs like $\mathbf{while}$-loops, the automaton traverses the input tree and performs multiple passes over subtrees, visiting the internal nodes of the tree many times. We then translate the two-way alternating tree automaton to an equivalent (one-way) nondeterministic top-down tree automaton by adapting results from [33, 53] to our setting. Finally, we check emptiness of the intersection between this top-down automaton and the grammar automaton . The definitions for trees and the relevant automata are standard, and we refer the reader to [14] and to our technical report [30].

### Tree Automaton for Program Trees

Every program can be represented as a tree whose leaves are labeled with basic statements like $\text {``}x :=y\text {''}$ and whose internal nodes are labeled with constructs like $\mathbf{while}$ and $\mathbf{seq}$ (an alias for the sequencing construct ‘$\mathbf ;$’), which have sub-programs as children. Essentially, we represent the set of programs generated by an input grammar $\mathcal {G}$ as a regular set of program trees, accepted by a nondeterministic top-down tree automaton . The construction of  mimics the standard construction for tree automata that accept *parse trees* of context free grammars. The formalization of this intuition is straightforward, and we refer the reader to [30] for details. We note the following fact regarding the construction of the acceptor of program trees from a particular grammar $\mathcal {G}$.

#### Lemma 1

 has size $O(|\mathcal {G}|)$ and can be constructed in time $O(|\mathcal {G}|)$.   $\square$

### Tree Automaton for Simulating Executions

We now discuss the construction of the two-way alternating tree automaton  that underlies our synthesis procedure. A two-way alternating tree automaton consists of a finite set of states and a transition function that maps tuples (*q*, *m*, *a*) of state, incoming direction, and node labels to positive Boolean formulas over pairs $(q',m')$ of next state and next direction. In the case of our binary program trees, incoming directions come from $\{D,U_L,U_R\}$, corresponding to coming down from a parent, and up from left and right children. Next directions come from $\{U,L,R\}$, corresponding to going up to a parent, and down to left and right children.

The automaton  is designed to accept the set of all program trees that correspond to correct and coherent programs. This is achieved by ensuring that a program tree is accepted precisely when all executions of the program it represents are accepted by the word automaton  (Sect. 5.1). The basic idea behind  is as follows. Given a program tree *T* as input,  traverses *T* and explores all the executions of the associated program. For each execution $\sigma$,  keeps track of the state that the word automaton  would reach after reading $\sigma$. Intuitively, an accepting run of  is one which never visits the unique rejecting state of  during simulation.

We now give the formal description of , which works over the alphabet $\varGamma _V$ described in Sect. 5.3.

***States.*** Both the full set of states and the initial set of states for  coincide with those of the word automaton . That is, $Q^\textsf {cc}= Q^\textsf {exec}$ and $I^\textsf {cc}= \{q^\textsf {exec}_0\}$, where $q^\textsf {exec}_0$ is the unique starting state of .

***Transitions.*** For intuition, consider the case when the automaton’s control is in state *q* reading an internal tree node *n* with one child and which is labeled by $a = \text {``}\mathbf{while} (x = y)\text {''}$. In the next step, the automaton simultaneously performs two transitions corresponding to two possibilities: entering the loop after assuming the guard $\text {``}x=y\text {''}$ to be true and exiting the loop with the guard being false. In the first of these simultaneous transitions, the automaton moves to the left child $n{\cdot }L$, and its state changes to $q_1'$, where $q'_1 = \delta ^\textsf {exec}(q, \text {``}\mathbf{assume} (x=y)\text {''})$. In the second simultaneous transition, the automaton moves to the parent node $n{\cdot }{U}$ (searching for the next statement to execute, which follows the end of the loop) and changes its state to $q'_2$, where $q'_2 = \delta ^\textsf {exec}(q, \text {``}\mathbf{assume} (x\ne y)\text {''})$. We encode these two possibilities as a *conjunctive* transition of the two-way alternating automaton. That is, $\delta _1^\textsf {cc}(q, m, a) = \big ((q'_1, L) \wedge (q'_2, U) \big )$.

For every *i*, *m*, *a*, we have $\delta _i(q_\mathsf {reject}, m, a) = \bot$, where $q_\mathsf {reject}$ is the unique, absorbing rejecting state of . Below we describe the transitions from all other states $q \ne q_\mathsf {reject}$. All transitions $\delta _i(q, m, a)$ not described below are $\bot$.

***Transitions from the Root.*** At the root node, labeled by $\text {``}\mathbf{root} \text {''}$, the automaton transitions as follows:

$$\begin{aligned} \begin{aligned} \delta _1^\textsf {cc}(q, m, \mathbf{root} ) = {\left\{ \begin{array}{ll} (q, L) \text { if } m = D\\ \texttt {true} \,\, \text { otherwise } \end{array}\right. } \end{aligned} \end{aligned}$$

A two-way tree automaton starts in the configuration where *m* is set to $D$. This means that in the very first step the automaton moves to the child node (direction $L$). If the automaton visits the root node in a subsequent step (marking the completion of an execution), then all transitions are enabled.

***Transitions from Leaf Nodes.*** For a leaf node with label $a \in \varGamma _0$ and state *q*, the transition of the automaton is $\delta _0^\textsf {cc}(q, D, a) = (\delta ^\textsf {exec}(q,a), U)$. That is, when the automaton visits a leaf node from the parent, it simulates reading *a* in  and moves to the resulting state in the parent node.

***Transitions from*** ${ \text {``}\mathbf{while} \text {''}}$ ***Nodes.*** As described earlier, when reading a node labeled by $\text {``}\mathbf{while} (x\sim y)\text {''}$, where $\sim \, \in \{=, \ne \}$, the automaton simulates both the possibility of entering the loop body as well as the possibility of exiting the loop. This corresponds to a conjunctive transition:

$$\begin{aligned} \delta _1^\textsf {cc}(q, m, \text {``}\mathbf{while} (x\sim y)\text {''})&= (q', L\big ) \wedge \big (q'', U)\\ \textit{where } q'&= \delta ^\textsf {exec}(q, \text {``}\mathbf{assume} (x \sim y)\text {''})\\ \textit{and } q''&= \delta ^\textsf {exec}(q, \text {``}\mathbf{assume} (x \not \sim y)\text {''}) \end{aligned}$$

Above, $\not \sim$ refers to $\text {``}=\text {''}$ when $\sim$ is $\text {``}\ne \text {''}$, and vice versa. The first conjunct corresponds to the execution where the program enters the loop body (assuming the guard is true), and thus control moves to the left child of the current node, which corresponds to the loop body. The second conjunct corresponds to the execution where the loop guard is false and the automaton moves to the parent of the current tree node. Notice that, in both the conjuncts above, the direction in which the tree automaton moves does not depend on the last move *m* of the state. That is, no matter how the program arrives at a $\mathbf{while}$ statement, the automaton simulates both the possibilities of entering or exiting the loop body.

***Transitions from*** ${ \text {``}\mathbf{ite} \text {''}}$ ***Nodes.*** At a node labeled $\text {``}\mathbf{ite} (x \sim y)\text {''}$, when coming down the tree from the parent, the automaton simulates both branches of the conditional:The first conjunct in the transition corresponds to simulating the word automaton on the condition $x \sim y$ and moving to the left child, i.e. the body of the $\mathbf{then}$ branch. Similarly, the second conjunct corresponds to simulating the word automaton on the negation of the condition and moving to the right child, i.e. the body of the $\mathbf{else}$ branch.

Now consider the case when the automaton moves *up* to an $\mathbf{ite}$ node from a child node. In this case, the automaton moves up to the parent node (having completed simulation of the $\mathbf{then}$ or $\mathbf{else}$ branch) and the state *q* remains unchanged:***Transitions from*** ${ \text {``}\mathbf{seq} \text {''}}$ ***Nodes.*** In this case, the automaton moves either to the left child, the right child, or to the parent, depending on the last move. It does not change the state component. Formally,

$$\begin{aligned} \begin{aligned} \delta _2^\textsf {cc}(q, m, \text {``}\mathbf{seq} \text {''}) = {\left\{ \begin{array}{ll} (q, L) \text { if } m = D\\ (q, R) \text { if } m = U_L\\ (q, U) \text { if } m = U_R\end{array}\right. } \end{aligned} \end{aligned}$$

The above transitions match the straightforward semantics of sequencing two statements $s_1; s_2$. If the automaton visits from the parent node, it next moves to the left child to simulate $s_1$. When it finishes simulating $s_1$, it comes up from the left child and enters the right child to begin simulating $s_2$. Finally, when simulation of $s_2$ is complete, the automaton moves to the parent node, exiting the subtree.

The following lemma asserts the correctness of the automaton construction and states its complexity.

#### Lemma 2

 accepts the set of all program trees corresponding to correct, coherent programs. It has size , and can be constructed in $O(2^{poly (|V|)})$ time.   $\square$

### Synthesis Procedure

The rest of the synthesis procedure goes as follows. We first construct a nondeterministic  tree automaton  such that . An adaptation of results from [33, 53] ensures that  has size  and can be constructed in time $O(2^{2^{\text {poly}(|V|)}})$. Next we construct a top-down nondeterministic tree automaton  such that , with size  and in time . Finally, checking emptiness of  can be done in time . If non-empty, a program tree can be constructed.

This gives us the central upper bound result of the paper.

#### Theorem 6

The grammar-restricted synthesis problem for uninterpreted coherent programs is decidable in $2\mathsf {EXPTIME}$, and in particular, in time doubly exponential in the number of variables and  in the size of the input grammar. Furthermore, a tree automaton representing the set of *all* correct coherent programs that conform to the grammar can be constructed in the same time.    $\square$

### Matching Lower Bound

Our synthesis procedure is optimal. We prove a $2\mathsf {EXPTIME}$ lower bound for the synthesis problem by reduction from the $2\mathsf {EXPTIME}$-hard acceptance problem of *alternating* Turing machines (ATMs) with exponential space bound [12]. Full details of the reduction can be found in [30].

#### Theorem 7

The grammar-restricted synthesis problem for coherent uninterpreted programs is $2\mathsf {EXPTIME}$-hard.

## Further Results

In this section, we give results for variants of uninterpreted program synthesis in terms of transition systems, Boolean programs, and recursive programs.

### Synthesizing Transition Systems

Here, rather than synthesizing programs from grammars, we consider instead the synthesis of transition systems whose executions must belong to a regular set. Our main result is that the synthesis problem in this case is $\mathsf {EXPTIME}$-complete, in contrast to grammar-restricted program synthesis which is $2\mathsf {EXPTIME}$-complete.

**Transition System Definition and Semantics.** Let us fix a set of program variables *V* as before. We consider the following finite alphabet

$$\begin{aligned} \varSigma _V = \{ \text {``}x :=y\text {''}, \text {``}x :=f(\overline{z})\text {''}, \text {``}\mathbf{assert} (\bot )\text {''}, \text {``}\mathbf{check} (x=y)\text {''} \,\mid \, x, y, \in V,\,\overline{z} \in V^r \} \end{aligned}$$

Let us define $\varGamma _V \subseteq \varSigma _V$ to be the set of all elements of the form $\text {``}\mathbf{check} (x=y)\text {''}$, where $x,y \in V$. We refer to the elements of $\varGamma _V$ as *check* letters.

A (deterministic) transition system *TS* over *V* is a tuple $(Q, q_0, H, \lambda , \delta )$, where *Q* is a finite set of states, $q_0 \in Q$ is the initial state, $H \subseteq Q$ is the set of halting states, $\lambda : Q \rightarrow \varSigma _V$ is a labeling function such that for any $q \in Q$, if $\lambda (q) = \text {``}\mathbf{assert} (\bot )\text {''}$ then $q \in H$, and $\delta : (Q \setminus H) \rightarrow Q \cup (Q \times Q)$ is a transition function such that for any $q \in Q \setminus H$, $\delta (q) \in Q \times Q$ iff $\lambda (q) \in \varGamma _\textit{V}$.

We define the semantics of a transition system using the set of executions that it generates. A *(partial) execution* $\pi$ of a transition system $TS=(Q, q_0, H, \lambda , \delta )$ over variables *V* is a finite word over the induced execution alphabet $\varPi _{V}$ (from Sect. 3) with the following property. If $\pi = a_0 a_1 \ldots a_n$ with $n \ge 0$, then there exists a sequence of states $q_{j_0}, q_{j_1}, \ldots , q_{j_n}$ with $q_{j_0}=q_0$ such that ($0 \le i \le n$):

- If $\lambda (q_{j_i})\notin \varGamma _V$ then $a_i = \lambda (q_{j_i})$, and if $i < n$ then $q_{j_{i+1}} = \delta (q_{j_i})$.
- Otherwise $$ \begin{aligned} {\left\{ \begin{array}{ll} \text {either } &{}a_i = \text {``}\mathbf{assume} (x=y)\text {''} \text { and } i<n\Rightarrow q_{j_{i+1}} = \delta (q_{j_i})\downharpoonright _1, \\ \text {or } &{}a_i = \text {``}\mathbf{assume} (x \ne y)\text {''} \text { and } i<n\Rightarrow q_{j_{i+1}} = \delta (q_{j_i})\downharpoonright _2 \end{array}\right. } \end{aligned} $$

In the above, we denote pair projection with $\downharpoonright$, i.e., $(t_1, t_2) \downharpoonright _i = t_i$, where $i \in \{1,2\}$. A *complete execution* is an execution whose corresponding final state ($q_n$ above) is in *H*. For any transition system *TS*, we denote the set of its executions by $\textsf {Exec}(TS)$ and the set of its complete executions by $\textsf {CompExec}(TS)$. The notions of *correctness* and *coherence* for transition systems are identical to their counterparts for programs.

**The Transition System Synthesis Problem.** We consider transition system specifications that place restrictions on executions (both partial and complete) using two regular languages *S* and *R*. Executions must belong to the first language *S* (which is prefix-closed) and all complete executions must belong to the second language *R*. A specification is given as two deterministic automata  and  over executions, where  and . For a transition system *TS* and specification automata  and , whenever  and  we say that *TS* satisfies its (syntactic) specification. Note that this need not entail correctness of *TS*. Splitting the specification into partial executions *S* and complete executions *R* allows us, among other things, to constrain the executions of non-halting transition systems.

#### Definition 3

**(Transition System Realizability and Synthesis).** Given a finite set of program variables *V* and deterministic specification automata  (prefix-closed) and  over the execution alphabet $\varPi _V$, decide if there is a *correct*, coherent transition system *TS* over *V* that satisfies the specification. Furthermore, produce one if it exists.

Since programs are readily translated to transition systems (of similar size), the transition system synthesis problem seems, at first glance, to be a problem that ought to have similar complexity. However, as we show, it is crucially different in that it allows the synthesized transition system to have *complete information* of past commands executed at any point. We will observe in this section that the transition system synthesis problem is $\mathsf {EXPTIME}$-complete.

To see the difference between program and transition system synthesis, consider program skeleton *P* from Example 2 in Sect. 2. The problem is to fill the hole in *P* with either $\texttt {y} :=\texttt {T}$ or $\texttt {y} :=\texttt {F}$. Observe that when *P* executes, there are *two* different executions that lead to the hole. In grammar-restricted program synthesis, the hole must be filled by a sub-program that is executed *no matter how the hole is reached*, and hence no such program exists. However, when we model this problem in the setting of transition systems, the synthesizer is able to produce transitions that depend on how the hole is reached. In other words, it does not fill the hole in *P* with *uniform* code. In this sense, in grammar-restricted program synthesis, programs have *incomplete information* of the past. We crucially exploited this difference in the proof of $2\mathsf {EXPTIME}$-hardness for grammar-restricted program synthesis (see [30]). No such incomplete information can be enforced by regular execution specifications in transition system synthesis, and indeed the problem turns out to be easier: transition system realizability and synthesis are $\mathsf {EXPTIME}$-complete.

#### Theorem 8

Transition system realizability is decidable in time exponential in the number of program variables and polynomial in the size of the automata  and . Furthermore, the problem is $\mathsf {EXPTIME}$-complete. When realizable, within the same time bounds we can construct a correct, coherent transition system whose partial and complete executions are in  and , respectively.

### Synthesizing Boolean Programs

Here we observe corollaries of our results when applied to the more restricted problem of synthesizing Boolean programs.

In Boolean program synthesis we interpret variables in programs over the Boolean domain $\{T, F\}$, and we disallow computations of uninterpreted functions and the checking of uninterpreted relations. Standard Boolean functions like $\wedge$ and $\lnot$ are instead allowed, but note that these can be modeled using conditional statements. We allow for *nondeterminism* with a special assignment $\text {``}b :=\texttt {*}\text {''}$, which assigns *b* nondeterministically to *T* or *F*. As usual, a program is correct when it satisfies all its assertions.

Synthesis of Boolean programs can be reduced to uninterpreted program synthesis using two special constants *T* and *F*. Each nondeterministic assignment is modeled by computing a $\texttt {next}$ function on successive nodes of a linked list, accessing a nondeterministic value by computing $\texttt {key}$ on the current node, and assuming the result is either *T* or *F*. Since uninterpreted programs must satisfy assertions in all models, this indeed captures nondeterministic assignment. Further, every term ever computed in such a program is equivalent to *T* or *F* (by virtue of the interleaved $\mathbf{assume}$ statements), making the resulting program coherent. The $2\mathsf {EXPTIME}$ upper bound for Boolean program synthesis now follows from Theorem 6. We further show that, perhaps surprisingly, the $2\mathsf {EXPTIME}$ lower bound from Sect. 5 can be adapted to prove $2\mathsf {EXPTIME}$-hardness of Boolean program synthesis.

#### Theorem 9

The grammar-restricted synthesis problem for Boolean programs is $2\mathsf {EXPTIME}$-complete, and can be solved in time doubly-exponential in the number of variables and  in the size of the input grammar.   $\square$

Thus synthesis for coherent uninterpreted programs is no more complex than Boolean program synthesis, establishing decidability and complexity of a problem which has found wide use in practice—for instance, the synthesis tool Sketch solves precisely this problem, as it models integers using a small number of bits and allows grammars to restrict programs with holes.

### Synthesizing Recursive Programs

We extend the positive result of Sect. 5 to synthesize coherent recursive programs. The setup for the problem is very similar. Given a grammar that identifies a class of recursive programs, the goal is to determine if there is a program in the grammar that is coherent and correct.

The syntax of recursive programs is similar to the non-recursive case, and we refer the reader to [30] for details. In essence, programs are extended with a new function call construct. Proofs are similar in structure to the non-recursive case, with the added challenge of needing to account for recursive function calls and the fact that  becomes a (visibly) pushdown automaton rather than a standard finite automaton. This gives a $2\mathsf {EXPTIME}$ algorithm for synthesizing recursive programs; a matching lower bound follows from the non-recursive case.

#### Theorem 10

The grammar-restricted synthesis problem for uninterpreted coherent *recursive* programs is $2\mathsf {EXPTIME}$-complete. The algorithm is doubly exponential in the number of program variables and  in the size of the input grammar. Furthermore, a tree automaton representing the set of all correct, coherent recursive programs that conform to the grammar can be constructed in the same time.

## Related Work

The automata and game-theoretic approaches to synthesis date back to a problem proposed by Church [13], after which a rich theory emerged [9, 18, 32, 48]. The problems considered in this line of work typically deal with a system reacting to an environment interactively using a finite set of signals over an infinite number of rounds. Tree automata over infinite *trees*, representing strategies, with various infinitary acceptance conditions (Büchi, Rabin, Muller, parity) emerged as a uniform technique to solve such synthesis problems against temporal logic specifications with optimal complexity bounds [31, 38, 44, 45]. In this paper, we use an alternative approach from [35] that works on *finite* program trees, using two-way traversals to simulate iteration. The work in [35], however, uses such representations to solve synthesis problems for programs over a fixed finite set of Boolean variables and against LTL specifications. In this work we use it to synthesize coherent programs that have finitely many variables working over infinite domains endowed with functions and relations.

While decidability results for program synthesis beyond finite data domains are uncommon, we do know of some results of this kind. First, there are decidability results known for synthesis of tranducers with registers [29]. Transducers interactively read a stream of inputs and emit a stream of outputs. Finite-state tranducers can be endowed with a set of registers for storing inputs and doing only equality/disequality comparisons on future inputs. Synthesis of such transducers for temporal logic specifications is known to be decidable. Note that, although the data domain is infinite, there are no functions or relations on data (other than equality), making it a much more restricted class (and grammar-based approaches for syntactically restricting transducers has not been studied). Indeed, with uninterpreted functions and relations, the synthesis problem is undecidable (Theorem 1), with decidability only for coherent programs. In [11], the authors study the problem of synthesizing uninterpreted terms from a grammar that satisfy a first-order specification. They give various decidability and undecidability results. In contrast, our results are for programs with conditionals and iteration (but restricted to coherent programs) and for specifications using assertions in code.

Another setting with a decidable synthesis result over unbounded domains is work on strategy synthesis for linear arithmetic *satisfiability* games [17]. There it is shown that for a satisfiability game, in which two players (SAT and UNSAT) play to prove a formula is satisfiable (where the formula is interpreted over the theory of linear rational arithmetic), if the SAT player has a winning strategy then a strategy can be synthesized. Though the data domain (rationals) is infinite, the game consists of a finite set of interactions and hence has no need for recursion. The authors also consider reachability games where the number of rounds can be unbounded, but present only sound and incomplete results, as checking who wins in such reachability games is undecidable.

Tree automata techniques for accepting finite parse trees of programs was explored in [37] for synthesizing reactive programs with variables over finite domains. In more recent work, automata on finite trees have been explored for synthesizing data completion scripts from input-output examples [55], for accepting programs that are verifiable using abstract interpretations [54], and for relational program synthesis [56].

The work in [36] explores a decidable logic with $\exists ^* \forall ^*$ prefixes that can be used to encode synthesis problems with background theories like arithmetic. However, encoding program synthesis in this logic only expresses programs of finite size. Another recent paper [27] explores sound (but incomplete) techniques for showing unrealizability of syntax-guided synthesis problems.

## Conclusions

We presented foundational results on synthesizing coherent programs with uninterpreted functions and relations. To the best of our knowledge, this is the first natural decidable program synthesis problem for programs of arbitrary size which have iteration/recursion, and which work over infinite domains.

The field of program synthesis lacks theoretical results, and especially decidability results. We believe our results to be the first of their kind to fill this lacuna, and we find this paper exciting because it bridges the worlds of program synthesis and the rich classical synthesis frameworks of systems over finite domains using tree automata [9, 18, 32, 48]. We believe this link could revitalize both domains with new techniques and applications.

Turning to practical applications of our results, several questions require exploration in future work. First, one might question the utility of programs that verify only with respect to uninterpreted data domains. Recent work [10] has shown that verifying programs using uninterpreted abstractions can be extremely effective in practice for proving programs correct. Also, recent work by Mathur et al. [40] explores ways to add *axioms* (such as commutativity of functions, axioms regarding partial orders, etc.) and yet preserve decidability of verification. The methods used therein are compatible with our technique, and we believe our results can be extended smoothly to their decidable settings. A more elaborate way to bring in complex theories (like arithmetic) would be to marry our technique with the *iterative* automata-based software verification technique pioneered by work behind the Ultimate tool [23–26]; this won’t yield decidable synthesis, but still could result in *complete* synthesis procedures.

The second concern for practicality is the coherence restriction. There is recent work by Mathur et al. [41] that shows single-pass heap-manipulating programs respect a (suitably adapted) notion of coherence. Adapting our technique to this setting seems feasible, and this would give an interesting application of our work. Finally, it is important to build an implementation of our procedure in a tool that exploits pragmatic techniques for constructing tree automata, and the techniques pursued in [54–56] hold promise.

## Footnotes

1 Note, however, that both Sketch and SyGuS problems are defined using functions and relations that are interpreted using standard theories like arithmetic, etc., and hence of course do not have decidable synthesis.

2 We use superscripts ‘’ and ‘’ for word and tree automata, respectively.
